# Longitudinal study on birthweight and the incidence of endometrial cancer

**DOI:** 10.1038/sj.bjc.6604304

**Published:** 2008-03-18

**Authors:** F Xue, L A Hilakivi-Clarke, G L Maxwell, S E Hankinson, K B Michels

**Affiliations:** 1 Obstetrics and Gynecology Epidemiology Center, Department of Obstetrics, Gynecology and Reproductive Biology, Brigham and Women's Hospital, and Harvard Medical School, Boston, MA 02115, USA; 2 Georgetown University, Washington, DC, 20057 USA; 3 US Military Cancer Institute, Walter Reed Army Medical Center, Washington, DC, 20307 USA; 4 Channing Laboratory, Department of Medicine, Brigham and Women's Hospital, and Harvard Medical School, Boston, MA 02115, USA; 5 Department of Epidemiology, Harvard School of Public Health, Boston, MA 02115, USA

**Keywords:** endometrial cancer, birthweight, early life exposure, menopause, anthropometrics

## Abstract

From 1976 to 2004, we followed 71 751 participants of the Nurses' Health Study and identified 676 invasive endometrial cancer cases. Birthweight, assessed in 1992, was not associated with the incidence of endometrial cancer. No effect modification by menopausal status was observed, but statistical power to detect an interaction was limited.

Endometrial cancer is the commonest invasive gynecologic cancer in women in the United States, with nearly 40 000 new cases diagnosed each year (American Cancer Society, 2007). Birthweight and intrauterine exposures have been related to the risk of breast cancer (Michels and Xue, 2006), childhood leukaemia (McLaughlin *et al*, 2006) and testicular cancer (Michos *et al*, 2007). However, data are limited regarding the potential influence of early life exposures on endometrial cancer risk. Potential mechanisms for an association between high birthweight and increased breast cancer risk include exposure to elevated maternal pregnancy oestrogen levels (Petridou *et al*, 1990; Ekbom *et al*, 1992), and insulin-like growth factor-I levels (Yang and Yu, 2000; Ostlund *et al*, 2002), both of which also affect the development of endometrial cancer (Ayabe *et al*, 1997; Adami *et al*, 2002). Prenatal nutrition may regulate body size later in life by altering the number of adipocytes (Sayer and Cooper, 2005), reprogram metabolism or influence leptin resistance (Phillips *et al*, 1999; Breier *et al*, 2001). As obesity is an established risk factor of endometrial cancer for premenopausal and postmenopausal women (Adami *et al*, 2002), birthweight as a marker of prenatal nutrition may plausibly influence its development.

Using data from 28 years of follow-up of 71 751 women participating in the Nurses' Health Study (NHS), we examined the association between birthweight and incidence of endometrial cancer later in life.

## MATERIAL AND METHODS

The NHS was established in 1976, when 121 700 married registered nurses age 30–55 years replied to a baseline questionnaire and received questionnaires biennially by mail to update information on demographic, anthropometric, and life style factors, and on newly diagnosed disease. For the current analysis, we excluded women with missing birthweight data, prevalent cases of endometrial or other cancers, and women with hysterectomy at baseline. During follow-up, women were censored if they reported recently diagnosed *in situ* or invasive endometrial cancer, had a hysterectomy, died or were lost to follow-up.

In 1992, we asked participants to report their own birthweight with the following options: <2.500, 2500–<3182, 3182–<3863, 3863–<4545, ⩾4545 g. On each biennial questionnaire, participants were asked whether they had been newly diagnosed with endometrial cancer during the previous 2 years. The National Death Index was also routinely searched for deaths among women who did not respond to the questionnaires. For endometrial cancers reported by women or their next of kin for those who had died, permission was requested to review the relevant medical records. Study physicians reviewed all medical records and pathological reports to confirm their diagnosis. Cases included in this study were invasive epithelial endometrial cancers with stage greater than IA in the FIGO staging system.

At baseline and during follow-up, we inquired about a variety of personal characteristics including reproductive and life style factors, many being risk factors for endometrial cancer. Information on age, age at menarche, age at first birth and height was obtained at baseline in 1976. Other early life exposures including premature birth (2+ weeks premature) and duration of having been breast-fed were assessed in 1992, and birth order was queried in 2004. Weight at age 18 was assessed in 1980. Somatotype at age 5 and 10 was assessed in 1988 by asking participants to choose from nine diagrams that best depicted their figure outline at each age. Maternal vital status and family history of endometrial cancer was asked in 1996. Other covariates were inquired and updated during follow-up.

The association between birthweight and incidence of endometrial cancer was analysed using a Cox proportional hazards model. Three covariate-adjusted models were pursued. In the first model (model I), we adjusted only for family history of endometrial cancer and other early life exposures including birth order, duration of being breast-fed and premature birth, in addition to age. In the second (model II), we also included other established or potential risk factors for endometrial cancer. In the third (model III), we additionally adjusted for anthropometric factors, including somatotype at ages 5 and 10, BMI at age 18, and current BMI. Though covariate-adjusted models II and III have better goodness of fit due to additional adjustment for potential risk factors of endometrial cancer, these factors are subsequent to birthweight and could conceivably mediate the effect of birthweight on endometrial cancer risk. Therefore, adjusted model I would be the preferred model to estimate the overall effect of birthweight.

As birthweight was not assessed until 1992, we conducted a sensitivity analysis restricting follow-up to 1992–2004 and compared the results with the primary analysis using the entire follow-up. We also evaluated potential effect modification by menopausal status, anthropometric factors, and other early-life exposures.

## RESULTS

During 1 678 702 person-years of follow-up among 71 751 women, 676 cases of incident endometrial cancer were confirmed. Based on the eligible person-years, the follow-up rate for the entire cohort in our study from 1976 to 2004 was 88%. Women with higher birthweight were less likely to have been prematurely delivered and to be the first born of their mother, were more likely breastfed, more likely to have ever smoked, and were slightly taller, than women with lower birthweight (Table 1).

Birthweight was not associated with endometrial cancer (HR (95% CI)=1.14 (0.83–1.57), 1.10 (0.85–1.42), 1.14 (0.89, 1.46) for a birthweight of <2500, 2500–<3182, 3182–<3863 **g**, respectively, as compared to ⩾3863 **g**, *P* for trend=0.52) in the age-adjusted analysis (Table 2). Results did not differ substantially after adjusting for other potential confounders (covariate-adjusted models I–III) (Table 2). When restricting the analysis to follow-up from 1992 to 2004, the effect estimates did not change substantially from the primary analysis (Table 2). A test for interaction also did not suggest significant heterogeneity with regard to period of follow-up (1976–1992 *vs* 1992–2004) (*P*=0.80).

The association between birthweight and endometrial cancer did not vary significantly by menopausal status (*P*=0.41); however, statistical power was limited for premenopausal cases (Table 3). It also did not vary by somatotype at age five, somatotype at age 10, BMI at age 18, or current BMI (all P for interaction⩾0.32).

## DISCUSSION

In two previous prospective cohort studies, both from Sweden, the risk of endometrial cancer has been investigated in relation to birthweight. Based on 112 cases, the incidence of endometrial cancer in women with birthweight of ⩾4000 **g** was found to be almost half that of women with birthweight of <3000 **g** (HR=0.55 (95% CI 0.36–1.17)) (McCormack *et al*, 2005). Based on 73, no significant association was found between birthweight and endometrial cancer (HR=0.65 (95% CI 0.34–1.24) comparing the incidence in women with birthweight <2500 **g** to that in women >3000 **g**) (Lof *et al*, 2007). With more than triple the cases of all previous studies combined, results from the current study do not suggest an association.

Endometrial cancer involves cancerous growth of the endometrium. Unlike breast epithelium, in which terminal differentiation occurs largely during the first full-term pregnancy, the endometrial lining undergoes repeated division and differentiation throughout the reproductive life of a woman. Though birthweight has been related to a higher intrauterine exposure to oestrogen (Petridou *et al*, 1990) and IGF-I (Yang and Yu, 2000), the relation of birthweight to the profile of endogenous hormone later in life is less clear. In one study, birthweight was not associated with overall premenopausal sex hormone levels, but was inversely associated with luteal estrone and estrone sulphate (Tworoger *et al*, 2006). Similarly, birthweight was found to be only weakly associated with serum IGF-I levels in adulthood (Schernhammer *et al*, 2007).

The statistical power of this study would allow us to detect a modest association, if it exists. Many risk factors for endometrial cancer and other early life exposures were assessed and accounted for in the analysis. Though birthweight was queried 16 years after the start of follow-up, when compared with the analysis restricted to prospective follow-up, results including the entire follow-up of 28 years did not differ appreciably. We have previously compared self-reported birthweight with data derived from birth certificates and found self-reported birthweight to be highly reliable (*r*=0.74) (Troy *et al*, 1996).

Current research suggests that women can decrease their risk of endometrial cancer mainly through maintaining normal weight, modifying lifestyle, and optimal use of oral contraceptives and postmenopausal hormones (Adami *et al*, 2002). However, a large number of new cases of endometrial cancer, especially among younger women cannot be accounted for by known risk factors. Clarification of any association between early life exposures and endometrial cancer risk awaits further investigation.

## Figures and Tables

**Table 1:** Age-standardised distribution of demographic characteristics among 71 751 women according to birthweight throughout follow-up 1976–2004 (*N*=1 678 702 person-years)

	**Birthweight (g)**			
**Demographic characteristics**	**<2500**	**2500–<3182**	**3182–<3863**	**⩾3863**
Family history of endometrial cancer (%)	3.4	2.9	3.0	3.7
Premature birth (%)	35.6	2.9	0.7	0.3
First born (%)	40.6	39.2	37.9	29.1
Breast fed (%)	42.4	52.0	57.0	62.3
Age at menarche (mean) (years)	12.4	12.4	12.4	12.5
BMI at age 18 (Mean) (kg m^−2^)	21.1	21.0	21.5	21.9
Height (Mean) (cm)	162	163	165	166
Nulliparous (%)	7.0	6.9	6.4	7.5
Mean number of children among parous women	3.2	3.2	3.2	3.2
Premenopausal (%)	35.3	35.9	36.2	35.7
Ever smoked (%)	54.3	54.8	56.1	58.9
Current BMI (Mean) (kg m^−2^)	25.3	25.0	25.5	25.8

**Table 2:** Hazard ratio of endometrial cancer by birthweight among 71 751 participants of the Nurses' Health Study during follow-up from 1976–2004 and from 1992–2004

			**HR (95% CI)**			
**Birthweight (g)**	**Person-years**	**No. of cases**	**Age-adjusted**	**Covariate –adjusted I^a^**	**Covariate –adjusted II^b^**	**Covariate –adjusted III^c^**
*Follow-up 1976–2004*						
⩾3863	221515	84	1.0	1.0	1.0	1.0
3182–<3863	750698	307	1.14 (0.89–1.46)	1.15 (0.90–1.47)	1.14 (0.89–1.47)	1.18 (0.92–1.51)
2500–<3182	521674	208	1.10 (0.85–1.42)	1.10 (0.84–1.42)	1.08 (0.83–1.41)	1.15 (0.88–1.50)
<2500	184815	77	1.14 (0.83–1.57)	1.12 (0.79–1.57)	1.09 (0.77–1.54)	1.13 (0.79–1.60)
*P* for trend			0.52	0.63	0.74	0.48
						
*Follow-up 1992–2004*						
⩾3863	70871	54	1.0	1.0	1.0	1.0
3182–<3863	245954	207	1.16 (0.85–1.56)	1.16 (0.86–1.58)	1.16 (0.86–1.58)	1.19 (0.87–1.61)
2500–<3182	171030	123	0.99 (0.72–1.37)	1.00 (0.72–1.38)	0.98 (0.71–1.36)	1.03 (0.74–1.43)
<2500	60391	52	1.16 (0.79–1.71)	1.19 (0.79–1.81)	1.17 (0.77–1.78)	1.19 (0.78–1.82)
*P* for trend			0.91	0.87	0.96	0.79

a Hazard ratio (HR) and 95% confidence interval (CI) were adjusted for age (continuous), premature birth (yes, no), birth order (first, second, third, fourth or above), duration of being breast fed in infancy (no, ⩽3, 4–8, ⩾9 months), and family history of endometrial cancer (yes, no).

b HR and 95% CI were adjusted for age (continuous), premature birth (yes, no), birth order (first, second, third, fourth or above), duration of being breast fed in infancy (no, ⩽3, 4–8, ⩾9 months), and family history of endometrial cancer (yes, no), age at menarche (⩽10, 11, 12, 13, 14, 15+ years), current and past contraceptive use (never, past<5 years, past⩾5 years, current< 5years, current⩾5 years), parity (1,2,3,4+), age at first birth (⩽24, 25–30, 30+ years), age at last birth (⩽24, 25–29, 30–34, 35–40, >40 years), physical activity (<3, 3–8, 9–17, 18–26, 27–41, 42+ mets per week), cigarette smoking (never, 1–20, 21–40, 41+ pack-years), tamoxifen use (never, past and <5 years, past and ⩾5 years, current and <5 years, current and ⩾5 years), menopausal status (yes, no), age at menopause (<50, 50–52, 53+ years), and postmenopausal hormone use (never, past and <5 years, past and ⩾5 years, current and <5 years, current and ⩾5 years).

c HR and 95% CI were adjusted for age (continuous), premature birth (yes, no), birth order (first, second, third, fourth or above), duration of being breast fed in infancy (no, ⩽3, 4–8, ⩾9 months), and family history of endometrial cancer (yes, no), age at menarche (⩽10, 11, 12, 13, 14, 15+ years), current and past contraceptive use (never, past<5 years, past⩾5 years, current<5 years, current⩾5 years), parity (1,2,3,4+), age at first birth (⩽24, 25–30, 30+ years), age at last birth (⩽24, 25–29, 30–34, 35–40, >40 years), physical activity (<3, 3–8, 9–17, 18–26, 27–41, 42+ mets per week), cigarette smoking (never, 1–20, 21–40, 41+ pack-years), tamoxifen use (never, past and <5 years, past and ⩾5 years, current and <5 years, current and ⩾5 years), menopausal status (yes, no), age at menopause (<50, 50–52, 53+ years), postmenopausal hormone use (never, past and <5 years, past and ⩾5 years, current and <5 years, current and ⩾5 years), BMI (continuous in kg m^−2^), BMI at age 18 (continuous in kg m^−2^), and somatotype at age 5 and age at 10 (ranked from 1–9).

**Table 3:** Hazard ratio of endometrial cancer by birthweight among 71 751 participants of the Nurses' Health Study during follow-up from 1976–2004 stratified by menopausal status

			**HR (95% CI)**			
**Birthweight (g)**	**Person-years**	**No. of cases**	**Age-adjusted**	**Covariate –adjusted I^a^**	**Covariate –adjusted II^b^**	**Covariate –adjusted III^c^**
*Premenopausal*						
⩾3863	72535	14	1.0	1.0	1.0	1.0
3182–<3863	280538	43	0.77 (0.41–1.44)	0.79 (0.42–1.48)	0.90 (0.46–1.78)	0.95 (0.47–1.91)
2500–<3182	194984	31	0.85 (0.44–1.63)	0.79 (0.41–1.54)	0.87 (0.43–1.76)	0.95 (0.46–1.97)
<2500	67533	8	0.59 (0.24–1.42)	0.45 (0.17–1.19)	0.40 (0.14–1.15)	0.43 (0.14–1.28)
*P* for trend			0.37	0.18	0.17	0.27
						
*Postmenopausal*						
⩾3863	130992	70	1.0	1.0	1.0	1.0
3182–<3863	409653	264	1.21 (0.93–1.58)	1.21 (0.93–1.58)	1.20 (0.92–1.57)	1.23 (0.94–1.61)
2500–<3182	284140	170	1.12 (0.85–1.48)	1.12 (0.85–1.49)	1.10 (0.83–1.46)	1.17 (0.87–1.56)
<2500	102233	67	1.21 (0.86–1.69)	1.21 (0.84–1.74)	1.19 (0.82–1.72)	1.22 (0.84–1.77)
*P* for trend			0.46	0.48	0.58	0.38

a Hazard ratio (HR) and 95% confidence interval (CI) were adjusted for age (continuous), premature birth (yes, no), birth order (first, second, third, fourth or above), duration of being breast fed in infancy (no, ⩽3, 4–8, ⩾9 months), and family history of endometrial cancer (yes, no).

b HR and 95% CI were adjusted for age (continuous), premature birth (yes, no), birth order (first, second, third, fourth or above), duration of being breast fed in infancy (no, ⩽3, 4–8, ⩾9 months), and family history of endometrial cancer (yes, no), age at menarche (⩽10, 11, 12, 13, 14, 15+ years), current and past contraceptive use (never, past<5 years, past⩾5 years, current< 5years, current⩾5 years), parity (1,2,3,4+), age at first birth (⩽24, 25–30, 30+ years), age at last birth (⩽24, 25–29, 30–34, 35–40, >40 years), physical activity (<3, 3–8, 9–17, 18–26, 27–41, 42+ mets per week), cigarette smoking (never, 1–20, 21–40, 41+ pack–years), tamoxifen use (never, past and <5 years, past and ⩾5 years, current and <5 years, current and ⩾5 years). Age at menopause (<50, 50–52, 53+ years), and postmenopausal hormone use (never, past and <5 years, past and ⩾5 years, current and <5 years, current and ⩾5 years) were additionally adjusted for in the analysis of postmenopausal endometrial cancer.

c HR and 95% CI were adjusted for age (continuous), premature birth (yes, no), birth order (first, second, third, fourth or above), duration of being breast fed in infancy (no, ⩽3, 4–8, ⩾9 months), and family history of endometrial cancer (yes, no), age at menarche (⩽10, 11, 12, 13, 14, 15+ years), current and past contraceptive use (never, past<5years, past⩾5years, current<5 years, current⩾5 years), parity (1,2,3,4+), age at first birth (⩽24, 25–30, 30+ years), age at last birth (⩽24, 25–29, 30–34, 35–40, >40 years), physical activity (<3, 3–8, 9–17, 18–26, 27–41, 42+ mets per week), cigarette smoking (never, 1–20, 21–40, 41+ pack-years), tamoxifen use (never, past and <5 years, past and ⩾5 years, current and <5 years, current and ⩾5 years), BMI (continuous in kg m^−2^), BMI at age 18 (continuous in kg m^−2^), and somatotype at age 5 and age at 10 (ranked from 1–9). Age at menopause (<50, 50–52, 53+ years), and postmenopausal hormone use (never, past and <5 years, past and ⩾5 years, current and <5 years, current and ⩾5 years) were additionally adjusted for in the analysis of postmenopausal endometrial cancer.
